# Prognostic significance of Epstein-Barr virus infection in gastric cancer: a meta-analysis

**DOI:** 10.1186/s12885-015-1813-9

**Published:** 2015-10-24

**Authors:** Xuechao Liu, Jianjun Liu, Haibo Qiu, Pengfei Kong, Shangxiang Chen, Wei Li, Youqing Zhan, Yuanfang Li, Yingbo Chen, Zhiwei Zhou, Dazhi Xu, Xiaowei Sun

**Affiliations:** 1 State Key Laboratory of Oncology in South China, Collaborative Innovation Center for Cancer Medicine, Guangzhou, China; 2 Department of Gastric and Pancreatic Surgery, Sun Yat-sen University Cancer Center, 651# East Dongfeng Road, Guangzhou, 510000 Guangdong Province China

**Keywords:** Gastric cancer, Epstein-Barr virus, Prognosis, Meta-analysis

## Abstract

**Background:**

The prognostic significance of Epstein-Barr virus (EBV) infection in gastric cancer (GC) remains unclear. Recently, a number of studies have investigated the association between EBV infection and the prognosis of GC with controversial results. We therefore conducted a meta-analysis to assess its prognostic significance.

**Methods:**

PubMed and EMBASE were searched for studies up to October 1, 2014. We investigated the association between EBV infection with survival in patients with GC. The pooled hazard ratio (HR) and its 95 % confidence interval (CI) were calculated to evaluate risk.

**Results:**

A final analysis of 8,336 patients with GC from 24 studies was performed. Our analysis results indicated that the pooled HR was 0.67 (95 % CI: 0.55–0.79; Z = 11.18, *P <* 0.001). Subgroup analyses stratified by region revealed that the protective role of EBV infection only remained in the Asian population (HR: 0.62, 95 % CI: 0.48–0.75; *P <* 0.001). When stratified by study quality and statistical methodology, the protective role could also be identified in high quality studies (HR: 0.67, 95 % CI: 0.55–0.79) and in univariate analysis studies (HR: 0.62, 95 % CI: 0.50–0.74). There was no evidence of significant heterogeneity and publication bias.

**Conclusions:**

The presence of EBV has a favorable impact on GC patient’s survival, especially in an Asian population. Future updated studies, especially large-scale randomized controlled studies stratified by region, are warranted as validation studies.

**Electronic supplementary material:**

The online version of this article (doi:10.1186/s12885-015-1813-9) contains supplementary material, which is available to authorized users.

## Background

Gastric cancer (GC), one of the most common malignant tumors in the digestive tract, is the fourth most commonly diagnosed cancer and the second leading cause of cancer-related mortality worldwide [1]. Although the etiology of GC is still ambiguous, infectious agents have increasingly attracted attention as the mechanism of neoplastic transformation [2]. As we all know, Helicobacter pylori (*H. pylori*) is the major causative agent of GC [3]. Another infectious agent, Epstein-Barr virus (EBV) has also been found to be associated with GC [4–6]. EBV is a ubiquitous γ-herpes virus, which is grouped as a member of the herpesviridae family, subfamily gamma-Herpesvirinae, genus lymphocryptovirus [7, 8]. Since its discovery in tumor cells of Burkitt’s lymphoma in 1964 [9], EBV has been detected in a range of cancers, such as lymphoid neoplasms, nasopharyngeal, and gastric epithelial malignancies [10]. EBV-associated gastric carcinoma (EBVaGC) is defined by the presence of EBV in the GC cells, which represents about 9 % of GC worldwide [11–14]. Therefore, EBVaGC is identified as a distinct disease entity consisting of lymphoepithelioma-like carcinoma (LELC) and conventional adenocarcinoma [6, 15]. Though LELC has been reported to present a relatively favorable prognosis [16], the prognostic significance of EBVaGC is still controversial. A recent large-scale study from Huang SC et al. revealed no difference in survival between the EBVaGC cases and the EBV-negative cases [17]. Genitsch V et al. reported that there was no significant survival advantage for EBVaGC overall [18]. In addition, He Y et al. also drew consistent conclusion [19]. Considering that a pooled analysis including 13 studies revealed a protective role for EBV infection in the prognosis of GC [20], we conducted an extensive search for articles that evaluated the association between EBV and the outcome of GC. Here, a meta-analysis was performed to more precisely estimate the association between EBV infection and the prognosis of GC.

## Methods

### Search strategy and selection criteria

**Fig. 1:**
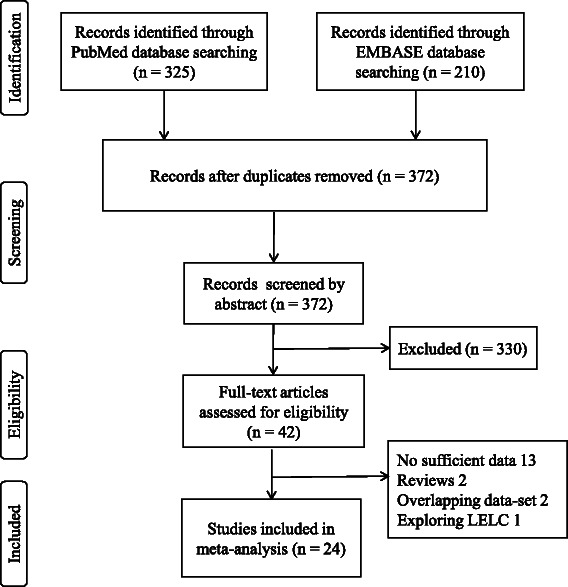
Flow chart of articles selection for meta-analysis. Abbreviations: LELC, lymphoepithelioma-like carcinoma

Two electronic databases (i.e., PubMed and EMBASE) were searched to explore studies (published before 1 October, 2014) that investigated the prognostic significance of EBV infection on the prognosis of GC. There were no geographic or language restrictions. Medical Subject Headings (MeSH) words used were the following keywords “Epstein-Barr virus”, “stomach neoplasms”, “gastric cancer”, “gastric carcinoma”, “prognosis” and “survival”. We examined the authors’ names and affiliations carefully to avoid duplicate data or overlapping articles. Abstracts of articles (*n =* 535) were checked independently by two investigators (XCL and JJL) to determine if full text articles should be obtained (Fig. 1), and disagreements were resolved by discussion with our research team.

Studies were eligible if survival was analyzed in GC patients stratified by EBV status. The primary outcome of interest was overall survival (OS). The meta-analysis was based on OS at 3 or 5 years that was extracted from published papers or original patient’s data. OS was defined as the time from GC diagnosis to death or last follow-up. The eligibility criteria of the studies were as follows: to present a proven diagnosis of GC in patients; to provide a sensitive and reliable method for detection of the existent status of EBV; to evaluate the correlation between EBV status and patients’ OS; to report a hazard ratio (HR) and 95 % confidence interval (CI) or sufficient date to estimate the HR and 95 % CI according to methods previously described by Parmar et al. [21, 22].

### Data extraction

Data were extracted by two investigators (XCL and JJL) independently using a predefined form. Discrepancies were resolved by discussion within our research team. The following data items were recorded from each study: first author, year of publication, time of follow-up, region, number of patients with positive and negative tumors, method of detection, positive rate, results of univariate and multivariate survival analyses, HRs and 95 % CIs. If the relevant information was unavailable in the articles, we emailed the corresponding author for additional data.

### Quality assessment

Study quality was assessed independently by two researchers (XCL and PFK) with the Newcastle-Ottawa quality assessment scale (Additional file 1: Table S1). Disagreement was resolved by discussion within our research team. Each study was assessed on three main categories: selection, comparability and outcome. The Newcastle-Ottawa Scale (NOS) scores ranged from 0 to 9; and a score ≥ 6 indicated good quality. As this was a meta-analysis, we did not include any humans and/or animals. Our study was approved by the Research Ethics Committee at the Cancer Center of Sun Yat-sen University.

### Statistical analysis

Our research adhered to the Preferred Reporting Items for Systematic Reviews and Meta-Analyses (PRISMA) guidelines (Additional file 2: Table S2) [23, 24]. The effect of EBV infection on OS was measured by HR and the corresponding 95 % CI. If the 95 % CI for the pooled HR did not overlap 1, the effect was considered as statistically significant. At first, a fixed-effects model (the inverse variance method) was used for calculating pooled HRs. When significant heterogeneity was detected across studies, a random-effects model (DerSimonian and Laird method) was selected. The existence of heterogeneity between studies was assessed using the Cochrane Q test and I^2^ statistic; with P_Q_ < 0.05 and I^2^ > 50 % considered to represent substantial heterogeneity between studies [25].

The HR of each study was estimated by various published methods [21, 22]. The most accurate method was to retrieve the HR and 95 % CI from the reported results. When the study did not report the 95 % CI, it was calculated by its P-value or O-E statistic (difference between numbers of observed and expected events). If the study only provided OS curves for the two groups, the HR estimate and its 95 % CI were reconstructed by extracting survival rates at specified times. In addition, there were three studies that only provided a risk ratio (RR) to evaluate the correlation between EBV status and patient OS rates. We selected the studies for further analysis with caution.

Publication bias was evaluated using a funnel plot, Begg’s test and Egger’s test. An asymmetric plot suggested possible publication bias. A two-sided p value < 0.05 was considered statistically significant for the Begg’s test and Egger’s test [26, 27]. Kaplan-Meier curves were read by Engauge Digitizer version 4.1 (http://digitizer.sourceforge.net). Statistical analysis was carried out using Stata software (version 12.0). All P values were two-sided and the significance level was 0.05.

## Results

### Study selection and characteristics

A total of 535 articles were identified from two electronic databases, of which 24 studies were included for the meta-analysis in accordance with the selection criteria (Fig. 1) [16, 17, 19, 20, 28–48]. The positive rate of EBV varied from 2.02 % [35] to 33.3 % [36] and the overall EBV positivity was 9.3 %. Among these studies, 13 studies were performed in Asian patients [17, 19, 28–38], five studies in European patients [39–43] and six studies in American patients [16, 44–48]. For all 24 studies, the presence of EBV in cancer cells was assessed by *in situ* hybridisation for EBV-encoded RNA (EBER), the gold standard assay for detecting latent infection. Though a part of excluded studies used other methods for the detection of EBV, for example polymerase chain reaction-enzyme immunoassay (PCR-EIA) [49], no studies were excluded just because of inappropriate detection method. With the exclusion of 3 studies that didn’t provide follow-up data [31, 44, 48], the estimated median follow-up time was 3.9 years. The total number of included patients was 8,336, ranging from 87 [41, 42] to 1,114 [32] patients per study. Table 1 summarizes characteristics of all inclusive studies.

**Table 1:** Characteristics of individual studies included in the meta-analysis

Study	Region	Year	EBV-positive/EBV-negative	Statistical methodology	HR estimation	Study quality score
Gonzalez CA	Europe	2003	4/83	UA	HR + 95 % CI	6/9
Chow WH	Europe	1999	11/76	UA	HR + 95 % CI	5/9
Kim RH	Asia	2010	18/229	UA	HR + 95 % CI	9/9
Gulley ML	Americas	1996	11/84	UA	HR + 95 % CI	8/9
Corvalán A	Americas	2005	22/71	UA	HR + 95 % CI	9/9
van Beek J	Europe	2004	41/525	UA	survival curves	8/9
He Y	Asia	2012	21/97	UA	HR + 95 % CI	7/9
Herrera-Goepfert R	Americas	2005	8/127	UA	HR + 95 % CI	5/9
Corvalan A	Americas	2001	27/118	UA	HR + 95 % CI	6/9
Koriyama C	Asia	2007	49/100	UA	HR + 95 % CI	8/9
Boysen T	Europe	2011	18/168	UA	HR + 95 % CI	7/9
Nakao M	Asia	2011	20/351	UA	HR + 95 % CI	5/9
Sukawa Y	Asia	2012	18/204	UA	HR + 95 % CI	7/9
Chiaravalli AM	Europe	2006	18/78	UA	RR + 95 % CI	7/9
Gao Y	Asia	2009	21/1018	UA	HR + 95 % CI	7/9
Kijima Y	Asia	2003	25/334	UA	HR + 95 % CI	9/9
Koriyama C	Asia	2002	64/128	MA	HR + 95 % CI	6/9
Park ES	Asia	2009	50/407	MA	HR + 95 % CI	7/9
Song HJ	Asia	2010	123/405	UA	HR + 95 % CI	8/9
Grogg KL	Americas	2003	7/103	UA	RR + 95 % CI	9/9
Zhao J	Asia	2012	80/631	UA	survival curves	7/9
Huang SC	Asia	2014	51/943	UA	HR + 95 % CI	8/9
Lee HS	Asia	2004	63/1051	UA	HR + 95 % CI	7/9
Truong CD	Americas	2009	12/223	MA	RR + 95 % CI	8/9

Abbreviations: *EBV* Epstein-Barr Virus, *HR* hazard ratio, *CI* confidence interval, *UA* univariate analysis, *MA* multivariate analysis

### Quality assessment and publication bias

The range of quality scores was from five to nine stars and the median quality score was seven. We defined the quality score as more than six to indicate a high quality study (see Additional file 2: Table S2). As shown in Table 1, 21 of 24 quality scores were categorized as high quality studies. The other three studies were categorized as low quality studies [37, 42, 47].

### Overall analysis

**Fig. 2:**
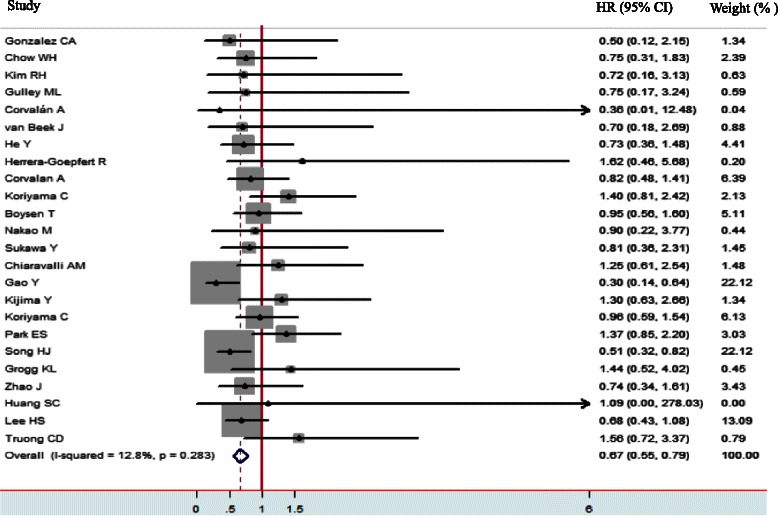
The forest plot demonstrates the effect sizes and 95 % confidence intervals (CIs) for each study and overall. Hazard ratios (HRs) with corresponding 95 % CIs of individual studies and pooled data for the association between Epstein-Barr virus-positive gastric cancer and overall survival. The forest plot demonstrates the effect sizes and 95 % CIs for each study and overall

**Fig. 3:**
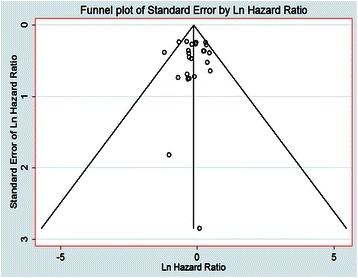
Funnel Plots for Studies. Funnel plots showing the relationship between the effect size of individual studies (standard error, horizontal axis) and the precision of the study estimate (hazard ratios for overall survival, vertical axis) for EBV

**Fig. 4:**
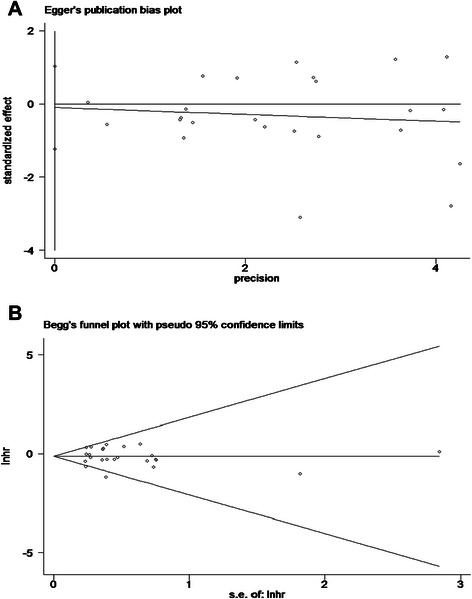
Publication bias plot for overall survival (**a**) Egger’s publication bias plot (**b**) Begg’s funnel plot

The main results of this meta-analysis and the heterogeneity test are presented in Table 2. Among the 24 studies eligible for the meta-analysis, 15 studies reported HRs and 95 % CIs [19, 28, 30–38, 41–43, 46, 47], three provided an RR and 95 % CI [16, 39, 48], two provided survival curves [34, 40] and four provided sufficient data to estimate the HR and 95 % CI [17, 29, 44, 45]. Figure 2 shows the forest plot of the effect sizes and 95 % CIs for each study and the overall value. The pooled HR for OS in GC patients was 0.67 (95 % CI: 0.55–0.79; Z = 11.18, *P <* 0.001) with a fixed-effects model. There was no significant evidence of heterogeneity across studies (I^2^ = 12.8 %, P_Q_ = 0.283). Investigation of publication bias by a funnel plot showed funnel plots was a slight lean (Fig. 3), but the judgments were subjective in nature. The Begg’s test (*P =* 0.655) and Egger’s test (*P =* 0.853) were used to further examine asymmetry of the funnel plot (Fig. 4). The P values of both tests were > 0.05 respectively, which suggested no evidence of publication bias.

**Table 2:** The prognostic significance of Epstein-Barr virus infection in gastric cancer by prespecified study characteristics in different subgroups

Stratified analysis	No. of Studies	Test of association				Test of heterogeneity		
		Pooled HR (95 % CI)	Z	P-value	Model	*X* ^2^	P-value	I^2^ (%)
Overall	24	0.67 (0.55,0.79)	11.18	<0.001	fixed-effects model	26.39	0.283	12.8
Region								
Asia	13	0.62 (0.48,0.75)	9.18	<0.001	fixed-effects model	19.65	0.074	38.9
Europe	5	0.87 (0.52,1.23)	4.87	<0.001	fixed-effects model	1.37	0.85	0
Americas	6	0.93 (0.53,1.34)	4.53	<0.001	fixed-effects model	1.76	0.881	0
Statistical methodology								
Univariate analysis	21	0.62 (0.50,0.74)	9.81	<0.001	fixed-effects model	18.52	0.553	0
Multivariate analysis	3	1.13 (0.76,1.50)	5.95	<0.001	fixed-effects model	1.36	0.506	0
Quality assessment								
High quality	21	0.67 (0.55,0.79)	10.93	<0.001	fixed-effects model	25.77	0.174	22.4
Low quality	3	0.83 (0.16,1.51)	2.41	0.016	fixed-effects model	0.4	0.819	0

Abbreviations: *EBV* Epstein - Barr virus, *HR* hazard ratio, *CI* confidence interval

### Subgroup and sensitivity analyses

Subgroup analyses were further performed to evaluate the effect of EBV infection on OS in GC patients more comprehensively, and there was no statistically significant heterogeneity or publication bias for all subgroup analyses. The concrete results were as follows: 1) When we stratified the studies by region, the pooled HR in Asia was 0.62 (95 % CI: 0.48–0.75; *P <* 0.001), the pooled HR in Europe was 0.87 (95 % CI: 0.52–1.23; *P <* 0.001), and in Americas 0.93 (95 % CI: 0.53–1.34; *P <* 0.001). After including three low-quality studies, the results of this subgroup analysis were similar (data not shown). 2) When stratified by study quality, the pooled HR for 21 high-quality studies was 0.67 (95 % CI: 0.55–0.79; *P <* 0.001) and the pooled HR for three low-quality studies was 0.83(95 % CI: 0.16–1.51; *P =* 0.016). 3) When further stratified by statistical methodology (univariate analysis results versus multivariate analysis results), the pooled HR for the univariate analysis results was 0.62 (95 % CI: 0.50–0.74; *P <* 0.001). However, the pooled HR for the multivariate analysis results was 1.13 (95 % CI: 0.76–1.50; *P <* 0.001), with no statistically significant differences.

Sensitivity analyses were carried out to determine if modification of the inclusion criteria for this meta-analysis affected the final results. First, sensitivity analyses to examine the influence of the individual data set to the pooled HR were performed by removing any one study individually and recalculating the pooled HR. The overall pooled HR and 95 % CI were not affected by a single study (data not shown), and the rang was from a low of 0.65 (95 % CI: 0.53–0.77; *P <* 0.001) to a high of 0.78 (95 % CI: 0.64–0.91; *P <* 0.001) via omission of the study by Park et al. [30] and the study by Gao et al. [35], respectively. Secondly, sensitivity analyses excluding data from the three studies only providing an RR and 95 % CI did not change the pooled HR (HR: 0.65; 95 % CI: 0.53–0.77; *P <* 0.001). Lastly, sensitivity analyses excluding the studies of which the HRs (95 % CI) were estimated from the survival curves did not alter the associations (HR: 0.67; 95 % CI: 0.55–0.79; *P <* 0.001).

## Discussion

In this study, we first overcame limits of size and region and showed that the presence of EBV has a favorable impact on GC patient survival.

Camargo MC et al. conducted a pooled analysis including 4,599 patients with GC from 13 studies in 2013. They found EBVaGC had a relative survival advantage [20]. The result was consistent with our study. However, there are some differences between these two studies. First, with the reports of new large sample studies, it is necessary to combine results to reach a more reliable conclusion. For example, a recent study including 994 stage I-III GC patients showed that the OS of EBV-positive patients with GC did not differ from that of EBV-negative patients [17]. He Y et al. also reported a similar conclusion [19]. In the current study, we included these new studies and had the largest cases series, a total of 8,336 patients with GC from 24 studies to explore the association to date. Second, in subgroup analysis, the conclusion of the subgroup analysis stratified by region was different [20]. There was an association between EBV infection and better survival in Asian patients. It was worth noting that, the protective role of EBV infection in European and American patients was not observed even after excluding low quality studies. However, Camargo MC et al. found that a survival advantage for EBVaGC was detected in Asia and Europe rather than Americas. Considering the limited number of European case series in the pooled analysis, we suggest our meta-analysis overcame limits of size and region to drive a more reliable conclusion. To date, the underlying reasons for these regional differences are still undefined. However, population differences in genetic factors may help explain part of the regional differences [50]. Studies have reported that EBVaGC displayed distinct clinical and genetic features. In EBVaGC, the prevalent types and variants of EBV in eastern countries were different from those in Latin American countries, suggesting that some EBV sequence variations might be geographically distributed [51, 52]. In addition, we speculated that there might be difference in the way of diagnosis and treatment between Asian and Western countries, though we didn’t find sufficient information from included studies. By now, the treament for GC is still ignored of the EBV status [53]. However, several promising therapeutic approaches are worthwhile to be further explored. A recent study from Hui KF et al. demonstrated that, the FDA (Food and Drug Administration)-approved Pan-histone deacetylase (HDAC) inhibitor romidepsin, which could potently induce EBV lytic cycle and mediate enhanced cell death with ganciclovir (GCV), might be applied for the treatment of EBVaGC [54]. Moreover, medical treatment with a demethylation agent may have particular merit in the therapy of EBVaGC, since methylation of the tumor suppressor gene is also a key abnormality in EBVaGC. Other potential medical treatment, such as proteosome inhibitor, antiviral drugs, inhibition of EBV-induced oncogenic cellular signaling pathways and EBV vaccines, may have an important role in the therapy of EBVaGC [11, 55]. Therefore, it is of interest whether difference in the way of treatment between Asian and Western countries impacts survival.

Furthermore, stratified subgroup analyses were performed by study quality and statistical methodology. We found the protective role of EBV infection in GC remained statistically significant in high quality studies and in univariate analysis results. However, the results of the multivariate analysis limited our conclusions. Considering that there has been only three multivariate analyses, our analysis should be viewed with caution.

Our meta-analysis found that patients with EBVaGC have a significantly better outcome than those with EBV-negative GC. Though many studies have been conducted to explore this phenomenon, the mechanisms underlying better outcomes of EBVaGC are still ambiguous, by far. Most cases of EBVaGC exhibit a histology rich in lymphocyte infiltration [39, 56], which may represent a relatively preferable prognosis in EBVaGC cases because of the improved anti-tumor immune response. In addition, genetic alteration and methylation of the tumor suppressor gene may be another key mechanism [57, 58]. It may be possible that, as reported in EBV-positive nasopharyngeal carcinoma, EBVaGC has a better prognosis in part because of better response to therapy [59, 60]. Further studies are needed to identify the mechanisms underlying this prognostic association.

Although we comprehensively evaluated the association between EBV and prognosis in GC with reasonable statistical methods, several limitations of the current meta-analysis should be addressed. First, we only explored the effect of EBV infection on OS in GC patients, and other factors that may contribute to the tumorigenesis of GC, such as genetic factors, environmental exposures and hereditary factors, were not considered. It is necessary to clarify the interactions between these factors and EBV infection in further studies. Secondly, it is difficult to acquire original data to remove other possible confounding factors, such as less p53 abnormal expression, higher expression of Human Interleukin 1 Beta (IL-1b) and so on. Thirdly, as we all know, the publicly accepted TNM system (7th UICC) is the gold standard to evaluate GC prognosis. In addition, it has been accepted that EBVaGC is typically located in non-antral subsites [11]. We are aware of the fact that differences in tumor location may impact survival, but the paucity of individual-level data on variables limits further study. Thus, our conclusion needs to be verified by studies of multivariate analysis adjusting for clinicopathological variables.

## Conclusions

To our knowledge, this study has the largest case series by far to explore the potential role of EBV in GC. We found that EBV infection has a favorable impact on GC patient survival, especially in the Asian population. Future studies, especially large-scale randomized controlled studies stratified by region, taking into account the classical well defined prognostic factors, are warranted as validation studies.

## Additional files

Additional file 1: Table S1. Newcastle-Ottawa Quality Assessment Scale. (PDF 36 kb)
Additional file 2: Table S2. PRISMA 2009 checklist in current meta-analysis. (DOC 70 kb)

## Abbreviations

EBV: Epstein-Barr virus
GC: Gastric cancer
HR: Hazard ratio
CI: Confidence interval
EBVaGC: EBV-associated gastric carcinoma
LELC: lLymphoepithelioma-like carcinoma
MeSH: Medical Subject Headings
OS: Overall survival
NOS: Newcastle-Ottawa Scale
PRISMA: Preferred Reporting Items for Systematic Reviews and Meta-Analyses
RR: Risk ratio
EBER: EBV-encoded RNA
PCR-EIA: Polymerase chain reaction-enzyme immunoassay
FDA: Food and Drug Administration
HDAC: Histone deacetylase
GCV: Ganciclovir
IL-1b: Human Interleukin 1 Beta
