# Supplementary material for: Prognostic significance of Epstein-Barr virus infection in gastric cancer: a meta-analysis
Source: BMC Cancer. 2015 Oct 24;15:782. doi: 10.1186/s12885-015-1813-9 (PMC4619309; doi:10.1186/s12885-015-1813-9)
Supplement: Additional file 1: Table S1. — Newcastle-Ottawa Quality Assessment Scale. (PDF 36 kb) [file 12885_2015_1813_MOESM1_ESM.pdf]

Appendix F. Newcastle-Ottawa Quality Assessment Scale

Assessment of quality of a cohort study – Newcastle-Ottawa Scale	
Selection (tick one box in each section)	
1. Representativeness of the intervention cohort<br>a) truly representative of the average, elderly, community-dwelling resident<br>b) somewhat representative of the average, elderly, community-dwelling resident<br>c) selected group of patients, e.g. only certain socio-economic groups/areas<br>d) no description of the derivation of the cohort	☐<br>☐<br>☐<br>☐
2. Selection of the non intervention cohort<br>a) drawn from the same community as the intervention cohort<br>b) drawn from a different source<br>c) no description of the derivation of the non intervention cohort	☐<br>☐<br>☐
3. Ascertainment of intervention<br>a) secure record (eg health care record)<br>b) structured interview<br>c) written self report<br>d) other / no description	☐<br>☐<br>☐<br>☐
4. Demonstration that outcome of interest was not present at start of study<br>a) yes<br>b) no	☐<br>☐
Comparability (tick one or both boxes, as appropriate)	
1. Comparability of cohorts on the basis of the design or analysis<br>a) study controls for age, sex, marital status<br>b) study controls for any additional factors (e.g. socio-economic status, education)	☐<br>☐
Outcome (tick one box in each section)	
1. Assessment of outcome<br>a) independent blind assessment<br>b) record linkage<br>c) self report<br>d) other / no description	☐<br>☐<br>☐<br>☐
2. Was follow up long enough for outcomes to occur<br>a) yes, if median duration of follow-up >= 6 month<br>b) no, if median duration of follow-up < 6 months	☐<br>☐
3. Adequacy of follow up of cohorts<br>a) complete follow up: all subjects accounted for<br>b) subjects lost to follow up unlikely to introduce bias: number lost <= 20%, or description of those lost suggesting no different from those followed<br>c) follow up rate < 80% (select an adequate %) and no description of those lost<br>d) no statement	☐<br>☐<br>☐<br>☐

NOS – CODING MANUAL FOR COHORT STUDIES

SELECTION

1) Representativeness of the Exposed Cohort (NB exposure = intervention)

Item is assessing the representativeness of exposed individuals in the community, not the representativeness of the study sample from some general population. For example, subjects derived from groups likely to contain exposed people are likely to be representative of exposed individuals, while they are not representative of all people the community.

Allocation of points as per rating sheet

2) Selection of the Non-Exposed Cohort

Allocation of points as per rating sheet

3) Ascertainment of Exposure

Allocation of points as per rating sheet

4) Demonstration That Outcome of Interest Was Not Present at Start of Study

In the case of mortality studies, outcome of interest is still the presence of a disease/ incident, rather than death. That is to say that a statement of no history of disease or incident earns a point.

COMPARABILITY

1) Comparability of Cohorts on the Basis of the Design or Analysis

Either exposed and non-exposed individuals must be matched in the design and/or confounders must be adjusted for in the analysis. Statements of no differences between groups or that differences were not statistically significant are not sufficient for establishing comparability. Note: If the relative risk for the exposure of interest is adjusted for the confounders listed, then the groups will be considered to be comparable on each variable used in the adjustment.

A maximum of 2 points can be allotted in this category.

OUTCOME

2) Assessment of Outcome

For some outcomes, reference to the medical record is sufficient to satisfy the requirement for confirmation. This may not be adequate for other outcomes where reference to specific tests or measures would be required.

a) Independent or blind assessment stated in the paper, or confirmation of the outcome by reference to secure records (health records, etc.)
b) Record linkage (e.g. identified through ICD codes on database records)
c) Self-report (i.e. no reference to original health records or documented source to confirm the outcome)
d) No description.

3) Was Follow-Up Long Enough for Outcomes to Occur

An acceptable length of time should be decided before quality assessment begins.

4) Adequacy of Follow Up of Cohorts

This item assesses the follow-up of the exposed and non-exposed cohorts to ensure that losses are not related to either the exposure or the outcome.

Allocation of points as per rating sheet
